# *Hyunsoonleella zeaxanthinifaciens* sp. nov., Isolated from Coastal Seawater of Jeju Island, Republic of Korea

**DOI:** 10.4014/jmb.2606.06040

**Published:** 2026-08-14

**Authors:** Ju-Hoon An, Jong Woo Hyeon, Mehwish Taj, Min-Hye Lee, Hanbi Kim, Sungjin Jung, Sang-Ah Lee, Man-Young Jung

**Affiliations:** 1 Interdisciplinary Graduate Program in Advanced Convergence Technology and Science, Jeju National University, Jeju 63243, Republic of Korea; 2 J2K Bio Co., Ltd., 50-3, Dureungyuri-ro, Ochang-eup, Cheongju-si, Chungcheongbuk-do 28104, Republic of Korea; 3 Department of Biology, Jeju National University, Jeju 63243, Republic of Korea; 4 Faculty of Biotechnology, College of Applied Life Sciences, Jeju National University, Jeju 63243, Republic of Korea; 5 Department of Biology Education, Jeju National University, Jeju 63243, Republic of Korea; 6 Jeju Microbiome Center, Jeju National University, Jeju, 63243, Republic of Korea

**Keywords:** *Hyunsoonleella zeaxanthinifaciens*, Novel species, *Hyunsoonleella*, Carotenoid, Zeaxanthin

## Abstract

A Gram-stain-negative, strictly aerobic, yellow-pigmented, non-motile, rod-shaped rod-shaped bacterium, designated J2K-J805^T^, was isolated from coastal seawater of Jeju Island, Republic of Korea. Strain J2K-J805^T^ grew at 15–37°C (optimum, 30°C), pH 6.0–8.0 (optimum, pH 7.0–7.5) and in the presence of 1–4% (w/v) NaCl (optimum, 2–3%) and was catalase-positive and oxidase-negative. The strain showed the highest 16S rRNA gene sequence similarity to *Hyunsoonleella flava* T58^T^ (98.4%). Phylogenetic and phylogenomic analyses based on the 16S rRNA gene and whole-genome sequences placed strain J2K-J805^T^ within the genus *Hyunsoonleella* as a distinct lineage. The OrthoANIu and digital DNA-DNA hybridization values between strain J2K-J805^T^ and *H. flava* T58^T^ were 87.7 and 34.5%, respectively, both below their respective thresholds for species delineation. The genome size and DNA G+C content were 3,825,400 bp and 34.9 mol%, respectively. The predominant respiratory quinone was MK-6, and the major cellular fatty acids were iso-C_15:1_ G, iso-C_15:0_ and iso-C_17:0_ 3-OH. The polar lipid profile comprised phosphatidylethanolamine, three unidentified aminolipids, two unidentified glycolipids and five unidentified lipids. High-performance liquid chromatography with photodiode-array detection identified the major yellow pigment as zeaxanthin, and the genome contained the carotenoid biosynthesis genes *crtB*, *crtI*, *crtY* and *crtZ* together with genes for isoprenoid precursor supply. On the basis of these polyphasic data, strain J2K-J805^T^ represents a novel species, for which the name *Hyunsoonleella zeaxanthinifaciens* sp. nov. is proposed. The type strain is J2K-J805^T^ (= KCTC 102498^T^ = JCM 38469^T^).

## Introduction

The genus *Hyunsoonleella*, a member of the family *Flavobacteriaceae* within the phylum *Bacteroidota*, was proposed by Yoon *et al*. in 2010, with *Hyunsoonleella jejuensis* as the type species [1]. At the time of writing, the genus comprises eight species with validly published and correct names (https://lpsn.dsmz.de/genus/hyunsoonleella) [2]. Members of the genus have been recovered mainly from marine and coastal environments, including seawater, coastal sediments and macroalgae, and are generally described as Gram-stain-negative, aerobic, yellow- to orange-pigmented, non-motile rods [1, 3-5].

Zeaxanthin is a xanthophyll carotenoid that is biosynthesized by a wide range of organisms, including plants, algae and microorganisms [6]. Numerous bacteria are known to produce zeaxanthin and other carotenoids, highlighting microorganisms as an important source of carotenoid diversity [7, 8]. Within the genus *Hyunsoonleella*, zeaxanthin has been reported as the major carotenoid of the type species *H. jejuensis* [1]. More broadly, many members of the family *Flavobacteriaceae* are yellow- to orange-pigmented, and recent genome-based and biochemical analyses indicate that marine *Flavobacteriaceae* commonly synthesize zeaxanthin through carotenoid biosynthetic routes linked to the mevalonate pathway [9].

In this study, we isolated a yellow-pigmented, zeaxanthin-producing strain, designated J2K-J805^T^, from coastal seawater of Jeju Island, Republic of Korea, and assessed its taxonomic position within the genus *Hyunsoonleella* using a polyphasic approach.

## Materials and Methods

### Isolation and Bacterial Strains

Strain J2K-J805^T^ was isolated from coastal seawater collected from Jongdal-ri, Jeju Island, Republic of Korea (33.510722° N, 126.906260° E). The seawater sample was serially diluted in artificial seawater (ASW; per liter: 20.0 g NaCl, 2.9 g MgSO_4_, 4.53 g MgCl_2_·6H_2_O, 0.64 g KCl, and 1.75 g CaCl_2_·2H_2_O), and aliquots were spread onto marine agar (MA; BD, USA) and incubated at 25°C for 5 days under aerobic conditions. Genomic DNA from colonies grown on MA was extracted using the AccuPrep Genomic DNA Extraction Kit (Bioneer, Republic of Korea) and 16S rRNA genes were PCR-amplified using the universal primers 27F (5′-AGA GTT TGA TCM TGG CTC AG-3′) and 1492R (5′-TAC GGY TAC CTT GTT ACG ACT T-3′). The resulting PCR amplicons were double-digested with HaeIII and HhaI, and representative PCR products showing identical fragment patterns were partially sequenced using the universal primer 340F (5′-CCT ACG GGA GGC AGC AG-3′), as described previously [10]. The 16S rRNA gene sequences were compared with those of all validly published type strains using the Nucleotide Similarity Search program (https://www.ezbiocloud.net/identify) [11]. On the basis of 16S rRNA gene sequence comparisons, a putative novel strain of the genus *Hyunsoonleella*, designated strain J2K-J805^T^, was selected for further phenotypic and phylogenetic analyses. The strain was routinely cultured aerobically on MA at 25°C for 2 days and was preserved at -80°C in marine broth (MB; BD) containing 15% (v/v) glycerol. For comparative phenotypic and chemotaxonomic analyses, three reference type strains, *Hyunsoonleella flava* T58^T^ (=KCTC 72081^T^), *H. jejuensis* CNU004^T^ (=KCTC 22242^T^ =DSM 21035^T^) and *H. ulvae* HU1-3^T^ (=KCTC 82511^T^), were obtained from the Korean Collection for Type Cultures (KCTC) and were cultured and characterized in parallel with strain J2K-J805^T^ under the same conditions.

### 16S rRNA Gene Sequencing and Phylogenetic Analysis

The 16S rRNA gene of strain J2K-J805^T^ was further sequenced using the universal primers 518R (5′-ATT ACC GCG GCT GCT GG-3′) and 805F (5′-GAT TAG ATA CCC TGG TAG TC-3′) to obtain a nearly complete 16S rRNA gene sequence [12]. The resulting 16S rRNA gene sequence was compared with those of validly published type strains using the Nucleotide Similarity Search program in EzBioCloud [11]. Multiple sequence alignments were performed using the ClustalW program implemented in MEGA X [13]. Phylogenetic trees were reconstructed using the neighbor-joining (NJ), maximum-parsimony (MP) and maximum-likelihood (ML) algorithms in MEGA X, and bootstrap values were calculated from 1,000 replications. The NJ tree was reconstructed using the Kimura two-parameter model [14], whereas the MP tree was obtained using the Subtree-Pruning-Regrafting (SPR) method [15]. The Tamura-Nei model was applied for ML analysis, and tree topology was searched using the Nearest-Neighbor-Interchange (NNI) method [16].

### Whole-Genome Sequencing and Assembly

Long-read genome sequencing of strain J2K-J805^T^ was performed in-house using the Oxford Nanopore MinION platform (UK). Raw sequencing reads were assessed using NanoPlot v1.46.2 to examine the total read count, total sequence yield, read-length distribution, read N50 and mean read quality [17]. The trimmed reads were assembled de novo using Flye v2.9.6-b1802 [18]. Genome completeness and contamination were estimated using CheckM2 v1.1.0 [19]. The whole-genome sequence of strain J2K-J805^T^ has been deposited in the GenBank database under accession number CM169901 and was annotated using the NCBI Prokaryotic Genome Annotation Pipeline (PGAP) [20]. A circular genome map was generated using a web-based platform for bacterial genome analysis and visualization, Proksee (https://proksee.ca) [21].

### Genome-Based Taxonomic and Phylogenomic Analyses

**Fig. 1:**
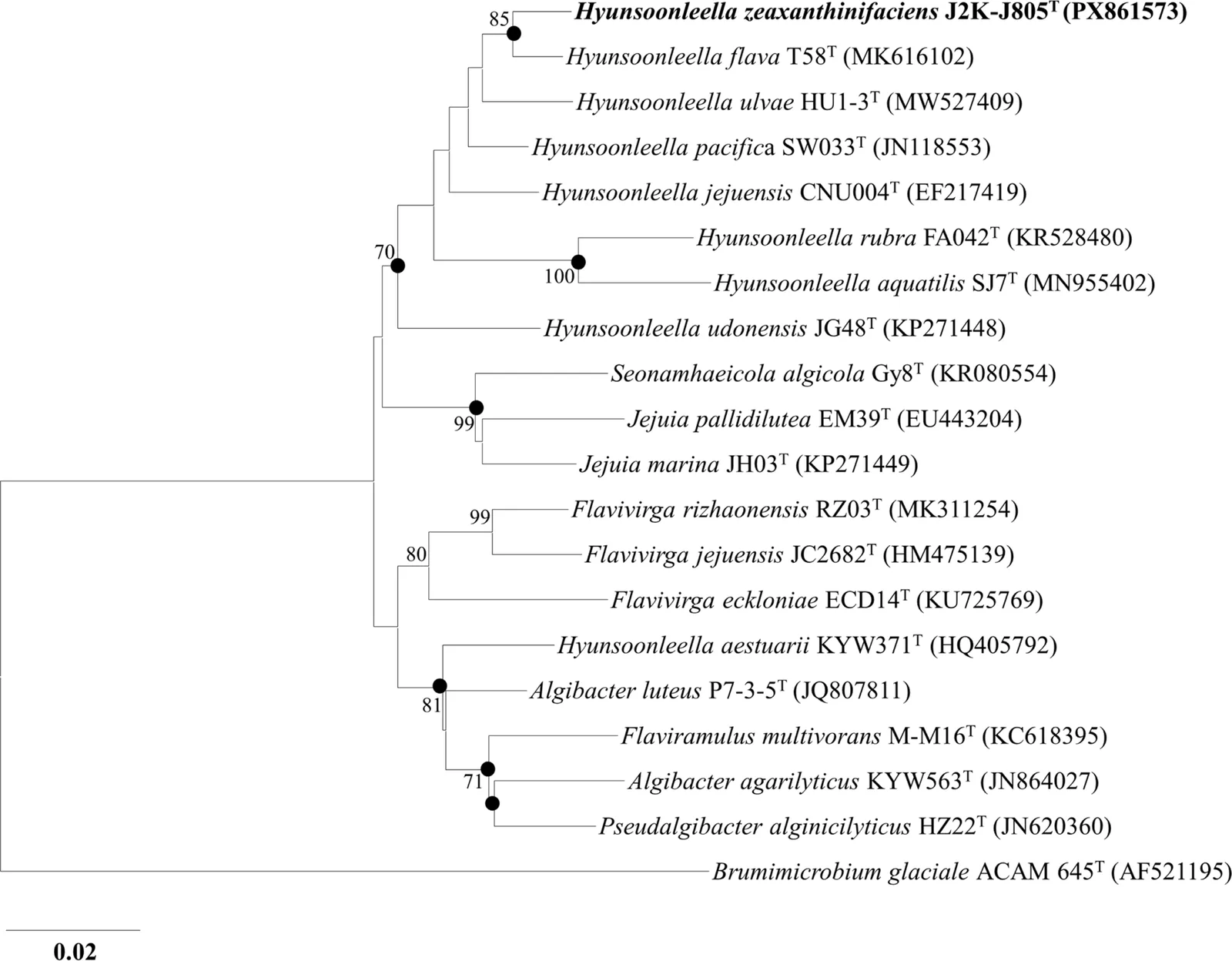
Neighbor-joining tree based on 16S rRNA gene sequences showing the phylogenetic relationships of strain J2K-J805^T^ and its closely related type strains. Bootstrap support values (>70%) are indicated at the nodes as percentages derived from 1,000 replicates. Filled circles (●) denote nodes that were also retrieved in the trees constructed using the maximum-parsimony and maximum-likelihood algorithms. *Brumimicrobium glaciale* ACAM 645^T^ (AF521195) was used as an outgroup. The scale bar equals 0.02 changes per nucleotide position.

**Fig. 2:**
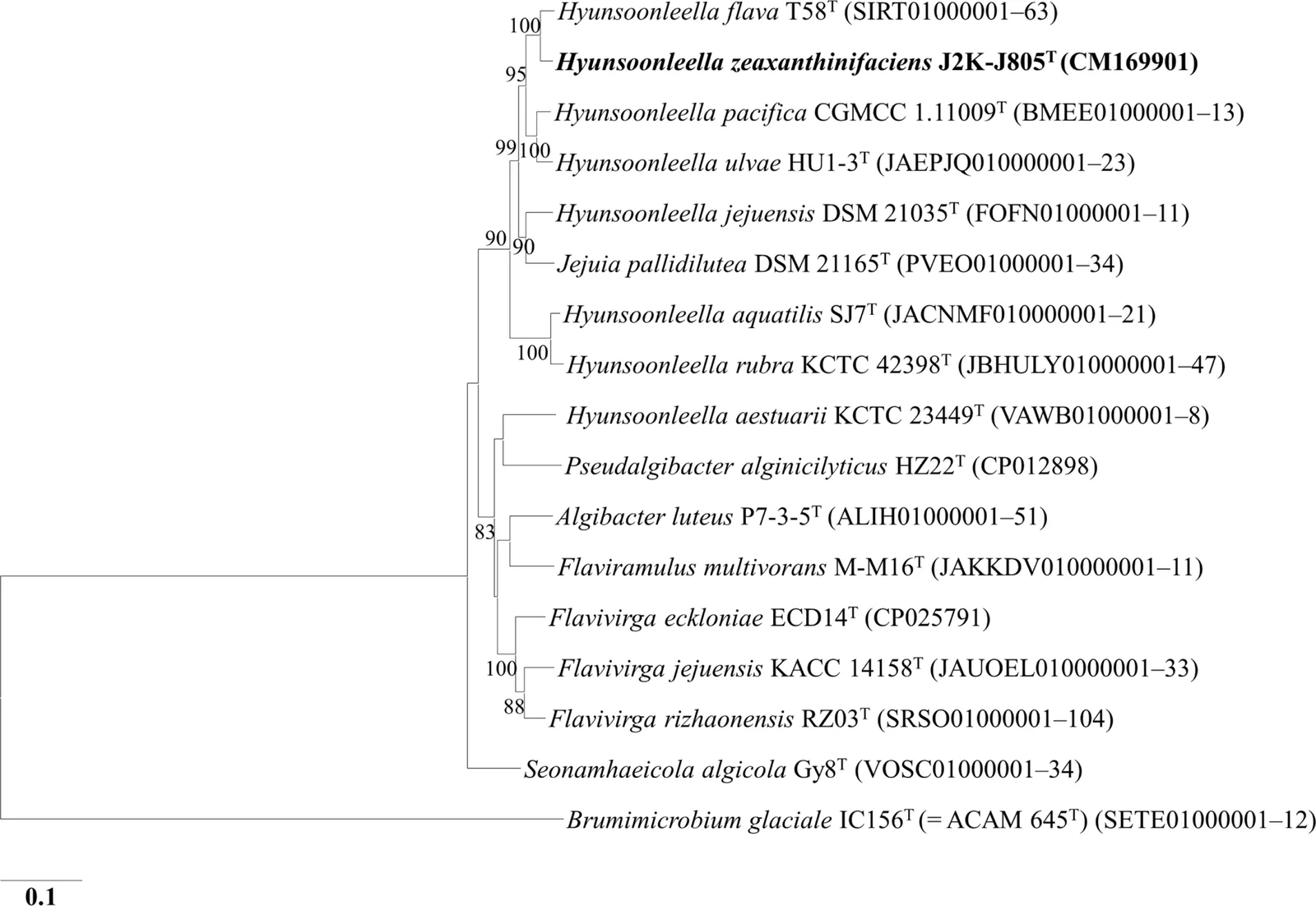
Phylogenomic tree showing the phylogenetic relationships of strain J2K-J805^T^ and its closely related type strains, based on the concatenated protein sequences of 120 single-copy marker genes (bac120 marker set). *H. udonensis*, *J. marina* and *A. agarilyticus* were excluded from this genome-based tree because no genome sequences for these species were available in the NCBI database. Bootstrap support values (>70%) are indicated at the nodes as percentages derived from 1,000 replicates. *Brumimicrobium glaciale* IC156^T^ (= ACAM 645^T^) was used as the outgroup. The scale bar equals 0.1 changes per amino acid position.

Genome-based taxonomic classification of strain J2K-J805^T^ was performed using Genome Taxonomy Database Toolkit (GTDB-Tk) v2.7.0 based on the concatenated protein sequences of 120 single-copy marker genes (bac120 marker set) [22]. All reference genomes used for the phylogenomic analysis were obtained from type strains. To harmonize taxon sampling between the two analyses, the phylogenomic tree was reconstructed using taxa included in the 16S rRNA gene-based tree for which type-strain genome sequences were publicly available. Because type-strain genome sequences for *Hyunsoonleella udonensis* JG48^T^, *Jejuia marina* JH03^T^, and *Algibacter agarilyticus* KYW563^T^ were not publicly available in the NCBI database at the time of analysis, these taxa were included only in the 16S rRNA gene-based analyses. *Brumimicrobium glaciale* IC156^T^ (= ACAM 645^T^) was used as the outgroup; its 16S rRNA gene sequence (AF521195) was used for the 16S rRNA gene-based analyses (Fig. 1 and Fig. S1), whereas its publicly available whole-genome sequence (WGS master accession SETE00000000.1; contigs SETE01000001–SETE01000012) was used for the genome-based analysis (Fig. 2). The taxa shared with the 16S rRNA gene-based phylogenetic tree were confirmed to represent the same type strains. The type-strain designations and available sequence accession numbers for the *Hyunsoonleella* taxa included in this study, the non-*Hyunsoonleella* neighboring taxa shared by both phylogenetic datasets, and the outgroup are provided in Table S1. An ML phylogenomic tree was reconstructed from the concatenated amino acid sequence alignment of these marker genes using MEGA X with the JTT matrix-based model [23], and bootstrap values were calculated from 1,000 replications. For species-level genomic comparisons, pairwise average nucleotide identity (ANI) values between strain J2K-J805^T^ and related *Hyunsoonleella* type strains were calculated using the Orthologous ANIu (OrthoANIu) tool available on the EzBioCloud server (https://www.ezbiocloud.net/tools/orthoaniu) [24]. Digital DNA-DNA hybridization values and their confidence intervals were estimated using the Genome-to-Genome Distance Calculator (GGDC 3.0; https://ggdc.dsmz.de/ggdc.php) using formula 2 (d_4_) [25]. Formula 2 was used as the representative estimate because it is independent of genome length and is suitable for comparisons involving draft genome assemblies.

### Comparative Functional Genome Analysis

**Fig. 3:**
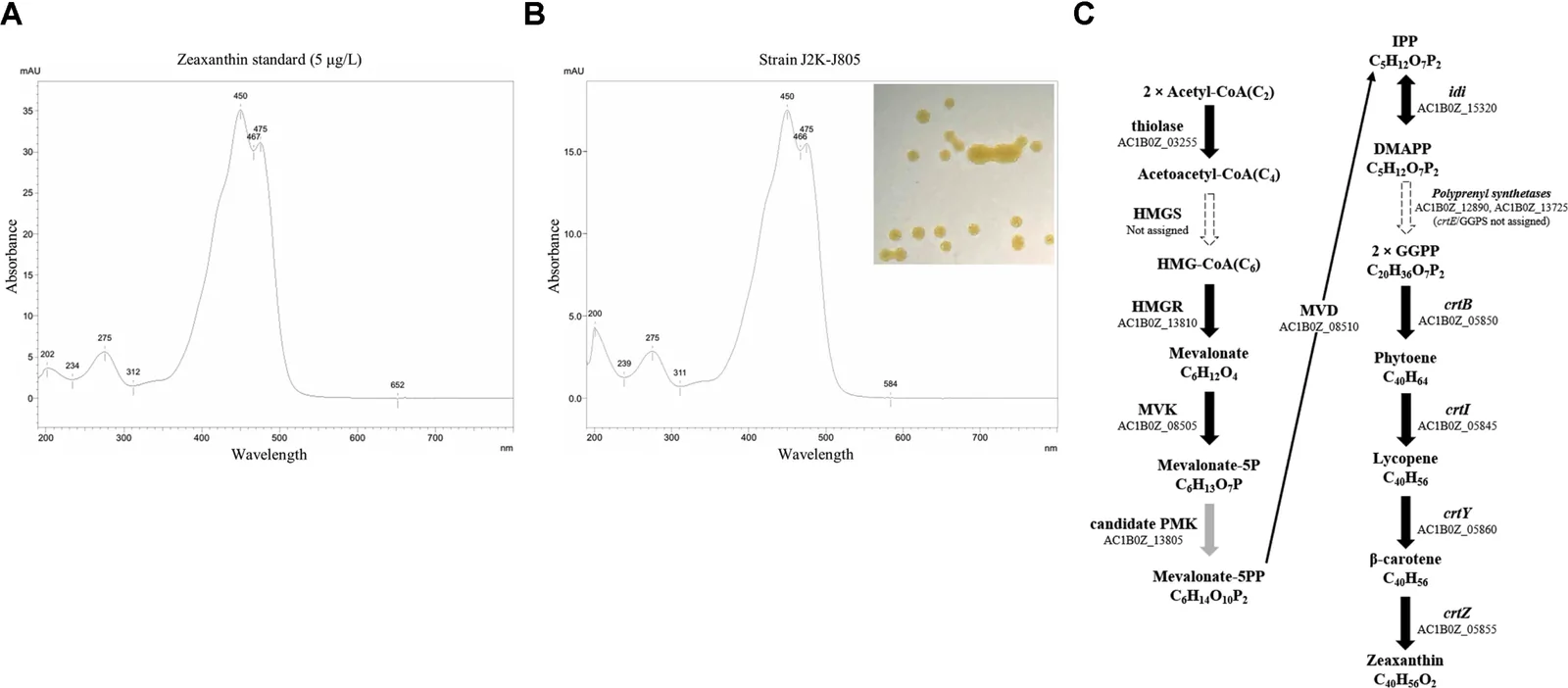
HPLC-PDA-based pigment identification and predicted zeaxanthin biosynthesis pathway of strain J2K-J805^T^. (A) PDA absorption spectrum of an authentic zeaxanthin standard (5 μg/L). (B) PDA absorption spectrum of the methanolic pigment extract of strain J2K-J805^T^, with yellow-pigmented colonies shown in the inset. (C) Proposed zeaxanthin biosynthesis pathway and MVA-related precursor supply inferred from genome annotation. Solid arrows indicate assigned reactions, whereas dashed or grey arrows indicate inferred or candidate steps. HMGS and a specific *crtE*/GGPS homolog were not assigned. Although a *dxs*-like gene was detected, the MEP pathway was not regarded as complete because downstream core MEP genes were not identified.

For comparative functional profiling, the assembled genome of strain J2K-J805^T^ and the genomes of three related *Hyunsoonleella* type strains were annotated using Bakta v1.11.4 with database version 6.0 [26], and the predicted protein sequences were analyzed using eggNOG-mapper v2.1.12 with the DIAMOND search strategy to assign Clusters of Orthologous Groups (COG) functional categories [27]. Carbohydrate-active enzymes (CAZy) were identified using the dbCAN3 server (https://pro.unl.edu/dbCAN2/blast.php) [28] and only genes annotated by all three tools (HMMER, DIAMOND and dbCAN-sub) were retained for further analysis. Genes involved in predicted zeaxanthin biosynthesis and isoprenoid precursor supply were identified from the PGAP annotation of the deposited genome and complementary Bakta annotations. The search included carotenoid biosynthesis genes (*crtB*, *crtI*, *crtY*, *crtZ* and *crtE*/GGPS), MVA-associated genes encoding acetyl-CoA C-acetyltransferase/thiolase, HMGS, HMGR, MVK, PMK, MVD and *idi*, and canonical MEP pathway genes (*dxs*, *dxr/ispC*, *ispD*, *ispE*, *ispF*, *ispG* and *ispH*). Publicly available NCBI PGAP feature tables of reference *Hyunsoonleella* genomes were also inspected for *crtE*/GGPS and prenyltransferase-related annotations. antiSMASH v8.0.4 was additionally used to inspect putative biosynthetic gene clusters [29]. Locus tags shown in Fig. 3C refer to the PGAP annotation of strain J2K-J805^T^.

### Phenotypic and Biochemical Characterization

Temperature-dependent growth was evaluated on MA at 4, 10, 15, 20, 25, 30, 37 and 40°C. Growth was assessed after 3 days, and cultures incubated at 4 and 10°C were further monitored for up to 2 weeks. To assess pH tolerance, growth was tested in marine broth (MB; BD) adjusted to pH 5.0–10.0 in 0.5-unit increments, using sodium citrate (pH 5.0), sodium phosphate (pH 6.0–7.0) and sodium carbonate (pH 8.0–10.0) as buffering agents, with pH adjusted post-autoclaving if needed [30]. Growth at different NaCl concentrations (0–7% at 1.0% intervals, w/v) was evaluated in marine broth (MB) prepared in the laboratory according to the BD formula. Anaerobic growth was assessed on MA at 25°C for 21 days using the GasPak Plus system (BBL, USA). Cell motility was evaluated using a semi-solid MB supplemented with 0.4% (w/v) agar [31]. Cell morphology was examined using transmission electron microscopy (JEM-1400Plus; JEOL, Japan). Gram staining was performed according to the standard method described previously [32]. Oxidase activity was determined using oxidase reagent (bioMérieux, France), and catalase activity was tested by observing bubble formation after the addition of 3% (v/v) hydrogen peroxide. Hydrolysis of starch, casein, Tween 20, and Tween 80 was assessed on MA according to the methods described previously [32, 33]. Additional biochemical characteristics of strain J2K-J805^T^ and reference strains were determined using API 20NE, API ZYM, and API 50CHB/E systems (bioMérieux), following the manufacturer’s instructions, except that cells resuspended in artificial seawater were used as inocula and the test strains were incubated at their optimal growth temperatures.

### Chemotaxonomic Characterization

For respiratory isoprenoid quinone analysis, strain J2K-J805^T^ was cultivated in MB at its optimal growth temperature until the exponential growth phase. Isoprenoid quinones were extracted as described previously [34] and analyzed using an HPLC system (LC-20A; Shimadzu, Japan) equipped with a diode array detector (SPD-M20A; Shimadzu) and a reversed-phase column (250 × 4.6 mm, Kromasil; Akzo Nobel, Sweden).

For cellular fatty acid analysis, strain J2K-J805^T^ and reference strains were cultured in MB at their optimal temperatures and harvested during the exponential growth phase. Fatty acid methyl esters were prepared using the standard MIDI protocol. The extracted fatty acid methyl esters were analyzed using gas chromatography (Hewlett Packard 6890, USA) and identified using the RTSBA6 database of the Microbial Identification System (Sherlock ver. 6.0B) [35].

Polar lipids were analyzed by two-dimensional TLC using cells harvested during the exponential growth phase as described previously [34]. Detection reagents included ethanolic molybdophosphoric acid (total polar lipids), ninhydrin (aminolipids), Dittmer-Lester reagent (phospholipids) and *α*-naphthol/sulfuric acid (glycolipids).

### Pigment Extraction and HPLC Analysis

Pigments were extracted with methanol from cell pellets harvested after cultivation for 2 days under routine conditions. The clarified methanolic extracts were analyzed using an HPLC system (LC-40 series; Shimadzu) equipped with a photodiode array detector (SPD-M40; Shimadzu) and a reversed-phase column (Zorbax SB-C18, 250 × 4.6 mm, 5 μm; Agilent Technologies, USA). Methanol/acetonitrile (90:10, v/v) was used as the mobile phase at a flow rate of 0.9 mL/min, and PDA spectra were recorded over 200–700 nm. Zeaxanthin analytical standard (Cat. No. 14681, Sigma-Aldrich) was used to prepare authentic standard solutions, which were analyzed under the same conditions for pigment assignment. The presence of flexirubin-type pigments was assessed by the KOH test [36].

## Results and Discussion

### Phylogenetic and Genome-Based Taxonomy

Strain J2K-J805^T^ showed the highest 16S rRNA gene sequence similarity to *H. flava* T58^T^ (98.4%). In the NJ tree, strain J2K-J805^T^ was placed within the *Hyunsoonleella* clade but formed a lineage separate from recognized *Hyunsoonleella* species (Fig. 1). Similar topologies were recovered in the MP and ML trees (Fig. S1). These results support the assignment of strain J2K-J805^T^ to the genus *Hyunsoonleella*. The genome-based ML phylogenomic tree placed strain J2K-J805^T^ within the genus *Hyunsoonleella* as a distinct genomic lineage, consistent with the 16S rRNA gene-based phylogeny (Fig. 2). The OrthoANIu values between strain J2K-J805^T^ and *H. flava* T58^T^, *H. jejuensis* DSM 21035^T^ and *H. ulvae* HU1-3^T^ were 87.72, 79.20 and 78.70%, respectively. The corresponding dDDH values were 34.5, 22.5 and 22.1%, respectively (Table S2). Although formula d_0_ gave a higher estimate for the comparison with *H. flava* T58^T^, all dDDH values calculated using the three formulae were below the 70% species boundary. The ANI and dDDH values were substantially below the generally accepted species delineation boundaries of 95–96% for ANI and 70% for dDDH [37], supporting the conclusion that strain J2K-J805^T^ represents a genomically distinct species within the genus *Hyunsoonleella*.

### Genomic Characteristics and Comparative Functional Profiles

The genome of strain J2K-J805^T^ was assembled into a single contig of 3,825,400 bp with an average sequencing coverage of 200× and a DNA G+C content of 34.9 mol% (Table 1). Genome completeness and contamination, estimated using CheckM2, were 99.98% and 0.01%, respectively, confirming the high quality of the assembly. Genome annotation predicted 3,316 genes, including 3,240 protein-coding genes, 47 RNA genes and 29 pseudogenes. By comparison, the genomes of the three related type strains ranged from 3,480 to 4,060 kb in size and contained 2,942–3,442 protein-coding genes. These results indicate that the genome size, DNA G+C content and coding capacity of strain J2K-J805^T^ were within the ranges observed for the compared *Hyunsoonleella* type strains. The circular genome map shows the distribution of coding sequences, RNA genes, selected isoprenoid- and carotenoid-related genes, GC content and GC skew (Fig. S2).

Comparative COG profiling showed broadly similar functional-category distributions across strain J2K-J805^T^ and the three reference type strains (Fig. S3), consistent with a conserved genome-wide functional composition at the genus level. In contrast, the carbohydrate-active enzyme (CAZy) repertoire differed in size among the compared strains. Strain J2K-J805^T^ encoded the fewest CAZy genes (131), comprising 60 glycoside hydrolases (GHs), 52 glycosyltransferases, 14 polysaccharide lyases, 4 carbohydrate esterases and 1 carbohydrate-binding module, whereas *H. flava* T58^T^, *H. jejuensis* DSM 21035^T^ and *H. ulvae* HU1-3^T^ encoded 140, 142 and 155 CAZy genes, respectively (Table 1). The difference was most pronounced for GHs, the largest CAZy class, which numbered 60 in strain J2K-J805^T^ versus 68–81 in the reference strains, with the macroalga-associated *H. ulvae* HU1-3^T^ containing the largest GH complement (81). Because GH content in marine *Flavobacteriaceae* is often associated with polysaccharide-degrading capacity and ecological niche, the comparatively reduced GH and overall CAZy repertoire of strain J2K-J805^T^ may reflect a more limited potential for complex carbohydrate utilization.

### Phenotypic and Biochemical Characteristics

Cells of strain J2K-J805^T^ were Gram-stain-negative, strictly aerobic, yellow-pigmented and non-motile rods measuring approximately 0.3–0.5 μm in width and 1.0–2.5 μm in length (Fig. S4). Many phenotypic characteristics of strain J2K-J805^T^, including esculin hydrolysis and enzyme activities such as catalase, alkaline phosphatase, esterase (C4) and esterase lipase (C8), were consistent with those of closely related *Hyunsoonleella* species (Table 2). In contrast, several characteristics, including nitrate reduction and hydrolysis of Tween 20, Tween 80 and gelatin, as well as enzyme activities such as trypsin and *β*-glucosidase, differentiated strain J2K-J805^T^ from other closely related *Hyunsoonleella* species (Table 2). Additionally, the API 50CHB/E acid-production profiles supported the phenotypic differentiation of strain J2K-J805^T^ from the reference *Hyunsoonleella* type strains (Table S3).

### Chemotaxonomic Characteristics

The predominant respiratory quinone of strain J2K-J805^T^ was MK-6, which has been reported as the major respiratory quinone in recognized *Hyunsoonleella* species [1, 3-5]. The predominant cellular fatty acids, each accounting for more than 10% of the total fatty acids, were iso-C_15:1_ G, iso-C_15:0_ and iso-C_17:0_ 3-OH (Table S4). The overall cellular fatty acid profile of strain J2K-J805^T^ was similar to those of closely related *Hyunsoonleella* strains, although strain-specific differences were observed in several fatty acid components (Table S4). The polar lipid profile comprised phosphatidylethanolamine, three unidentified aminolipids, two unidentified glycolipids and five unidentified lipids (Fig. S5). The polar lipid profile was generally comparable to those reported for closely related *Hyunsoonleella* strains [4, 5]. However, two unidentified glycolipids were only detected in strain J2K-J805^T^. The presence of MK-6, phosphatidylethanolamine and characteristic fatty acid components was consistent with the chemotaxonomic framework of the genus *Hyunsoonleella*. These data therefore support the genus-level affiliation of strain J2K-J805^T^, while the complete fatty acid and polar lipid profiles complement the genomic and phenotypic evidence used for species differentiation.

### Pigment Identification and Genomic Potential for Zeaxanthin Biosynthesis

Strain J2K-J805^T^ formed yellow-pigmented colonies, and no flexirubin-type pigment was detected by the KOH test. HPLC-PDA analysis of the methanolic pigment extract revealed a dominant peak at 6.229 min, which fell within the retention-time range of authentic zeaxanthin standards analyzed under the same conditions (6.204–6.229 min) (Table S5). The PDA spectrum of this peak showed absorption maxima near 450 and 475 nm and was consistent with that of the zeaxanthin standard (Fig. 3A and B), supporting assignment of the major yellow pigment as zeaxanthin; pigment identity was therefore based on the retention-time window and the matching PDA spectrum rather than on peak area. The genome of strain J2K-J805^T^ contained carotenoid biosynthesis genes *crtB*, *crtI*, *crtY* and *crtZ*, which are associated with the predicted conversion of geranylgeranyl diphosphate (GGPP) to zeaxanthin (Fig. 3C). The idi gene, mediating interconversion of isopentenyl diphosphate (IPP) and dimethylallyl diphosphate (DMAPP), was also present. Several MVA-associated genes were detected, including genes encoding thiolase, HMGR, MVK, a candidate PMK/GHMP kinase and MVD. Although a *dxs*-like gene was identified, the downstream MEP genes *dxr/ispC*, *ispD*, *ispE*, *ispF*, *ispG* and *ispH* were not detected; thus, a complete MEP pathway was not reconstructed. The presence of a *dxs*-like gene alone does not indicate an operational MEP pathway, because its product, 1-deoxy-D-xylulose 5-phosphate, is also a common precursor for thiamine and pyridoxal (vitamin B_6_) biosynthesis [38]; *dxs* is therefore commonly retained even in bacteria that rely on the MVA pathway for isoprenoid precursor supply. These findings suggest that C_5_ isoprenoid precursors for carotenoid biosynthesis may be supplied mainly through an MVA-related route, consistent with a recent report showing that marine *Flavobacteriaceae* can produce zeaxanthin using MVA-derived precursors [9]. HMGS and a canonical *crtE*/geranylgeranyl diphosphate synthase (GGPS) homolog were not assigned in the current annotation, and these steps were therefore treated as inferred or candidate reactions in Fig. 3C. A similar annotation pattern was observed in the examined reference *Hyunsoonleella* genome assemblies, in which no specific *crtE*/GGPS assignment was recovered, whereas polyprenyl synthetase- or prenyltransferase-related genes were present. Together, these genomic features support the predicted capacity of strain J2K-J805^T^ to synthesize zeaxanthin, while indicating that part of the upstream precursor-supply route remains unresolved.

## Taxonomic Conclusion

The 16S rRNA gene phylogeny, genome-based phylogeny, genomic relatedness, phenotypic characteristics and chemotaxonomic properties consistently support the placement of strain J2K-J805^T^ within the genus *Hyunsoonleella*. The OrthoANIu and dDDH values between strain J2K-J805^T^ and closely related *Hyunsoonleella* type strains were substantially below the accepted species delineation thresholds. In addition, strain J2K-J805^T^ could be differentiated from recognized *Hyunsoonleella* species by a combination of phenotypic and chemotaxonomic characteristics. On the basis of these combined results, strain J2K-J805^T^ is considered to represent a novel species of the genus *Hyunsoonleella*, for which the name *Hyunsoonleella zeaxanthinifaciens* sp. nov. is proposed.

### Description of *Hyunsoonleella zeaxanthinifaciens* sp. nov.

*Hyunsoonleella zeaxanthinifaciens* (ze.a.xan.thi.ni.fa’ci.ens. N.L. neut. n. *zeaxanthinum*, zeaxanthin; L. part. *faciens*, making, producing; N.L. part. adj. *zeaxanthinifaciens*, zeaxanthin-producing).

Cells are Gram-stain-negative, strictly aerobic, non-flagellated and non-motile rods, approximately 0.3–0.5 μm wide and 1.0–2.5 μm long. Colonies grown on marine agar are yellow. Growth occurs at 15–37°C (optimum, 30°C), at pH 6.0–8.0 (optimum, pH 7.0–7.5) and in the presence of 1–4% (w/v) NaCl (optimum, 2–3%). Catalase-positive and oxidase-negative. Nitrate reduction to nitrite is observed, whereas D-glucose fermentation and indole production do not occur. Esculin, Tween 20, Tween 80 and gelatin are hydrolyzed, whereas starch and casein are not hydrolyzed. Assimilation of D-glucose, D-mannose, L-arabinose, D-mannitol, D-maltose, potassium gluconate, capric acid, adipic acid, malic acid, trisodium citrate, phenylacetic acid and *N*-acetyl-glucosamine is negative. Alkaline phosphatase, esterase (C4), esterase lipase (C8), leucine arylamidase, valine arylamidase, cystine arylamidase, *α*-chymotrypsin, acid phosphatase, naphthol-AS-BI-phosphohydrolase, *α*-glucosidase, trypsin and *β*-glucosidase activities are positive, but arginine dihydrolase, urease, lipase (C14), *α*-galactosidase, *β*-glucuronidase, *α*-mannosidase, *α*-fucosidase, *β*-galactosidase and N-acetyl-*β*-glucosaminidase activities are negative. Acids are produced from D-mannose, amygdalin, D-lactose, salicin, D-melezitose, D-cellobiose, D-melibiose, glycogen, D-xylose, D-galactose, D-glucose, L-rhamnose, esculin, D-maltose, starch, *β*-gentiobiose and potassium 5-ketogluconate. The predominant respiratory quinone is MK-6. The predominant cellular fatty acids are iso-C_15:1_ G, iso-C_15:0_ and iso-C_17:0_ 3-OH. The polar lipid profile comprises phosphatidylethanolamine, three unidentified aminolipids, two unidentified glycolipids and five unidentified lipids. The major yellow pigment is zeaxanthin.

The type strain is J2K-J805^T^ (= KCTC 102498^T^ = JCM 38469^T^), isolated from coastal seawater collected at Jongdal-ri, Jeju Island, Republic of Korea. The DNA G+C content is 34.9 mol%. The GenBank accession numbers for the 16S rRNA gene and genome sequences are PX861573 and CM169901, respectively.

## Supplemental Materials

Supplementary data for this paper are available on-line only at http://jmb.or.kr.



## Figures and Tables

**Table 1:** Comparisons of genomic characteristics of strain J2K-J805^T^ and closely related *Hyunsoonleella* type strains.

Characteristic^†^	1	2	3	4
Genome status	Complete	Draft	Draft	Draft
No. of contigs	1	63	11	23
Total genome size (kb)	3,825	3,808	3,480	4,060
Average genome coverage	200×	290×	268×	270×
G+C content (mol%)	34.9	35.0	34.5	33.9
No. of total genes	3,316	3,368	2,996	3,504
No. of protein-coding genes	3,240	3,302	2,942	3,442
No. of total RNA genes	47	50	46	51
No. of rRNA genes	6	4	5	3
No. of tRNA genes	37	42	37	44
No. of pseudogenes	29	16	8	11
No. of total CAZy^‡^ genes	131	140	142	155
Glycoside hydrolase	60	71	68	81
Glycosyltransferase	52	46	51	57
Polysaccharide lyase	14	17	17	11
Carbohydrate esterase	4	4	4	4
Carbohydrate-binding module	1	2	2	2

Taxa: 1, strain J2K-J805^T^ (CM169901); 2, *H. flava* T58^T^ (SIRT01000001–63); 3, *H. jejuensis* DSM 21035^T^ (FOFN01000001–11); 4, *H. ulvae* HU1-3^T^ (JAEPJQ010000001–23).

^†^Genomic characteristics, except for CAZy genes, were derived from the in-house PGAP annotation generated in this study for taxon 1 and were extracted from the NCBI RefSeq database for taxa 2–4.

^‡^CAZy, carbohydrate-active enzymes..

**Table 2:** Differential phenotypic characteristics of strain J2K-J805^T^ and closely related *Hyunsoonleella* species.

Characteristic	1	2	3	4
Colony color	Yellow	Yellow	Yellow	Pale yellow
Growth range of:				
Temperature (°C)	15–37	15–37	15–37	15–37
pH	6.0–8.0	6.0–8.0	6.5–7.5	6.5–7.5
NaCl (%, w/v)	1–4	1–5	1–5	1–4
Nitrate reduction to nitrite	+	–	–	+
Glucose fermentation	–	+	–	+
Hydrolysis of:				
Starch	–	+	–	+
Tween 20	+	+	–	–
Tween 80	+	–	+	+
Casein	–	+	–	+
Gelatin	+	+	+	–
Assimilation of:				
D-Mannose	–	–	–	+
*N*-Acetyl-glucosamine	–	–	–	+
Enzyme activities of:				
Oxidase	–	+	+	+
Trypsin	+	+	+	–
*β*-Galactosidase	–	–	–	+
*β*-Glucosidase	+	+	+	–
*N*-Acetyl-*β*-glucosaminidase	–	–	+	–

Taxa: 1, strain J2K-J805^T^; 2, *H. flava* T58^T^ (=KCTC 72081^T^); 3, *H. jejuensis* CNU004^T^ (=KCTC 22242^T^ =DSM 21035^T^); 4, *H. ulvae* HU1-3^T^ (=KCTC 82511^T^). All data were obtained from this study. All strains are strictly aerobic and catalase-positive, hydrolyze esculin, and are positive for alkaline phosphatase, esterase (C4), esterase lipase (C8), leucine arylamidase, valine arylamidase, cystine arylamidase, *α*-chymotrypsin, acid phosphatase, naphthol-AS-BI-phosphohydrolase and *α*-glucosidase activities. All strains are negative for the following characteristics: motility, reduction of nitrite to nitrogen, indole production, assimilation of D-glucose, L-arabinose, D-mannitol, D-maltose, potassium gluconate, capric acid, adipic acid, malic acid, trisodium citrate and phenylacetic acid, enzyme activities of arginine dihydrolase, urease, lipase (C14), *α*-galactosidase, *β*-glucuronidase, *α*-mannosidase and *α*-fucosidase. Symbols: +, positive; –, negative.
