# Supplementary material for: Hyunsoonleella zeaxanthinifaciens sp. nov., Isolated from Coastal Seawater of Jeju Island, Republic of Korea
Source: J Microbiol Biotechnol. 2026 Aug 14;36:e2606040. doi: 10.4014/jmb.2606.06040 (PMC13506255; doi:10.4014/jmb.2606.06040)
Supplement: Supplementary file 1 [file jmb-36-e2606040-supple.pdf]

J. Microbiol. Biotechnol. 2026. 36: e2606040

https://doi.org/10.4014/jmb.2606.06040

Supplementary Figures and Tables

Hyunsoonleella zeaxanthinifaciens sp. nov., isolated from coastal seawater of Jeju Island, Republic of Korea

Ju-Hoon An[1#], Jong Woo Hyeon[1,2#], Mehwish Taj[1], Min-Hye Lee[3], Hanbi Kim[1], Sungjin Jung[4], Sang-Ah Lee[1,4,6] and Man-Young Jung[1,5,6*]

[1]*Interdisciplinary Graduate Program in Advanced Convergence Technology and Science, Jeju National University, Jeju 63243, Republic of Korea*

[2]*J2K Bio Co., Ltd., 50-3, Dureungyuri-ro, Ochang-eup, Cheongju-si, Chungcheongbuk-do 28104, Republic of Korea*

[3]*Department of Biology, Jeju National University, Jeju 63243, Republic of Korea*

[4]*Faculty of Biotechnology, College of Applied Life Sciences, Jeju National University, Jeju 63243, Republic of Korea*

[5]*Department of Biology Education, Jeju National University, Jeju 63243, Republic of Korea*

[6]*Jeju Microbiome Center, Jeju National University, Jeju, 63243, Republic of Korea*

[#]These authors contributed equally to this work.

*Corresponding author: myjung@jejunu.ac.kr

(A)

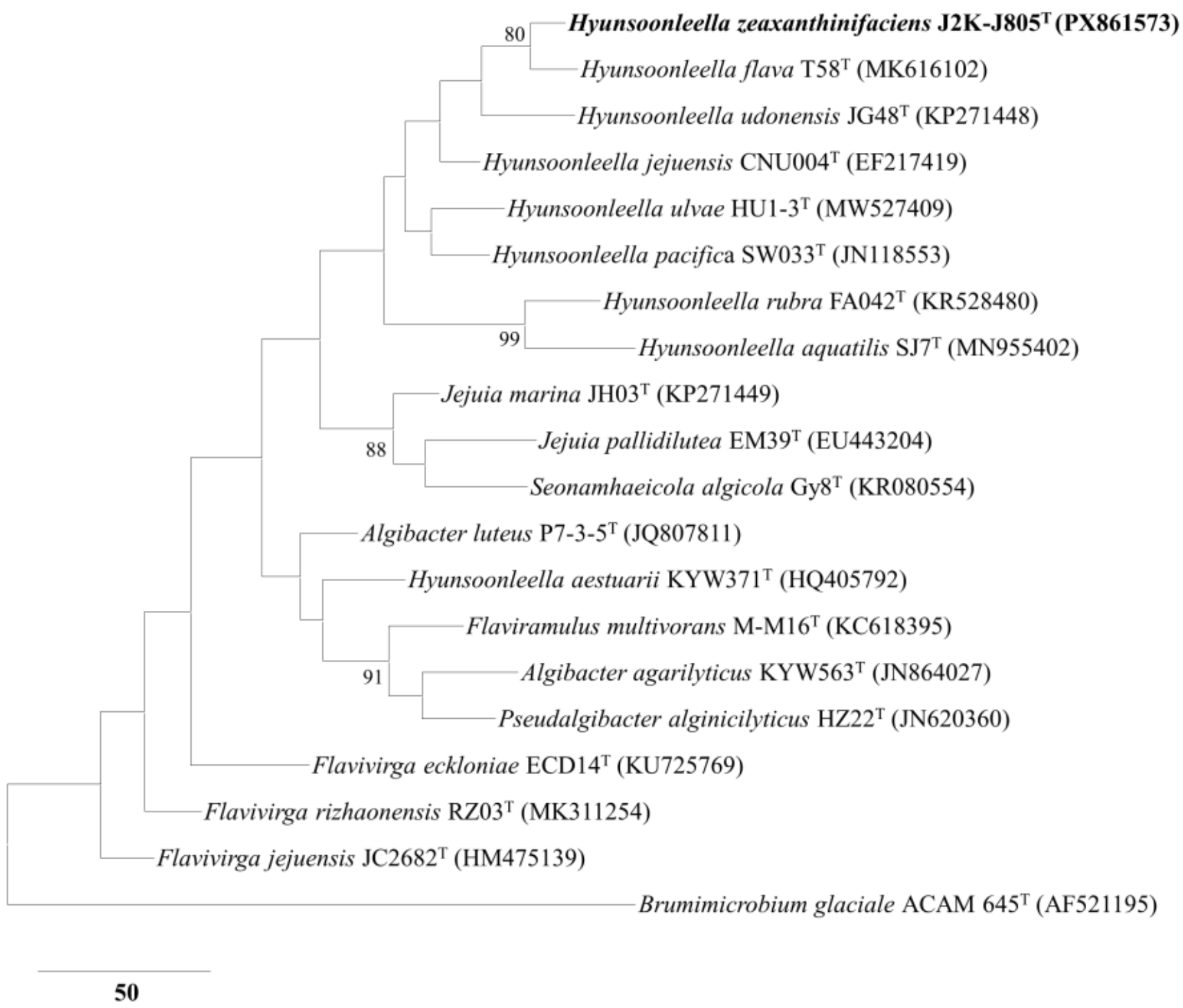

(B)

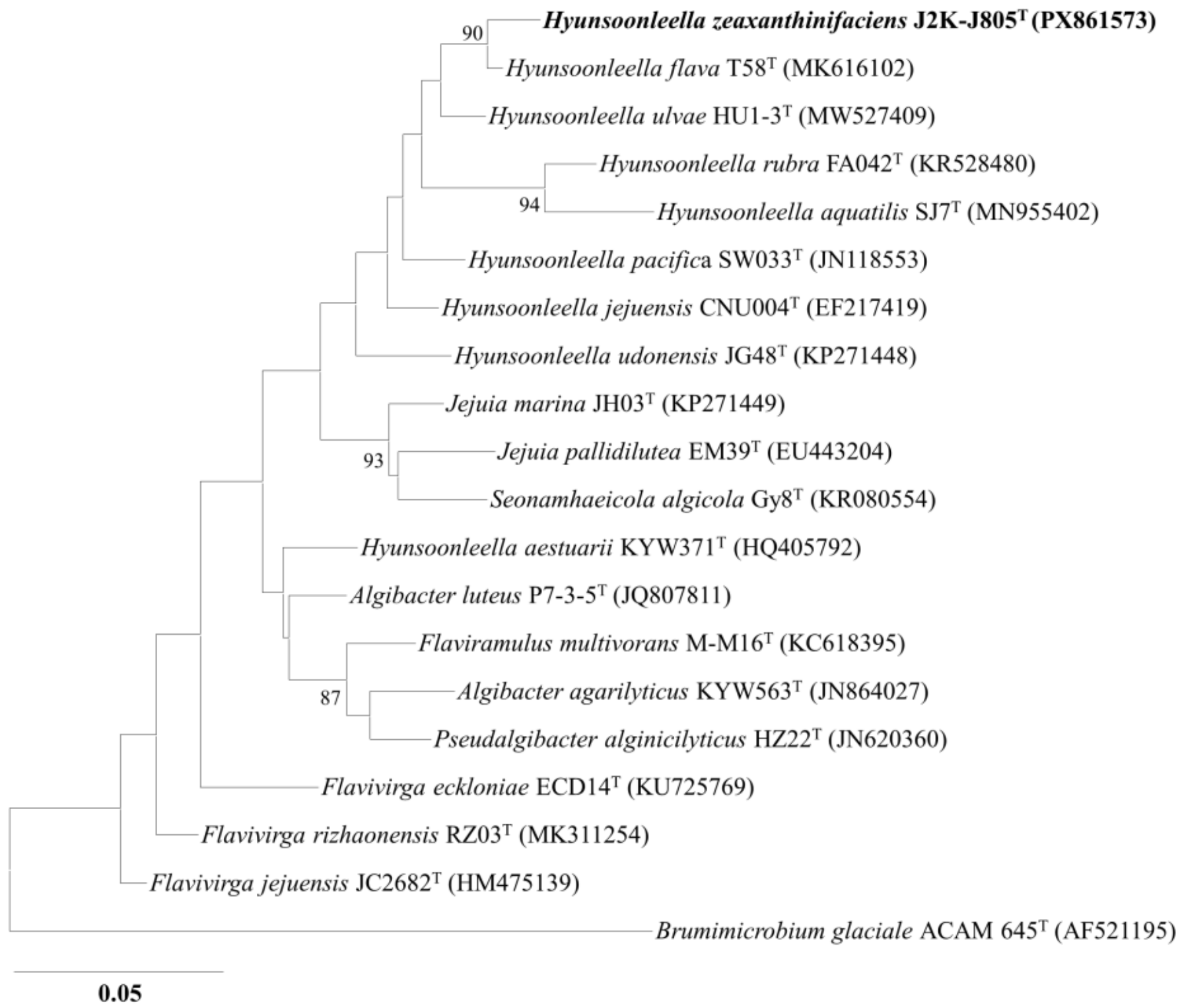

Fig. S1. Maximum-parsimony (MP; A) and maximum-likelihood (ML; B) phylogenetic trees showing the relationships of strain J2K-J805^{T} and their closely related taxa, based on 16S rRNA gene sequences. Bootstrap support values (>70%) are indicated at the nodes as

percentages derived from 1,000 replicates. *Brumimicrobium glaciale* ACAM 645^{T} (AF521195) was used as the outgroup. Scale bars represent the number of character changes across the full 16S rRNA gene sequence in the MP tree and the number of nucleotide substitutions per site in the ML tree.

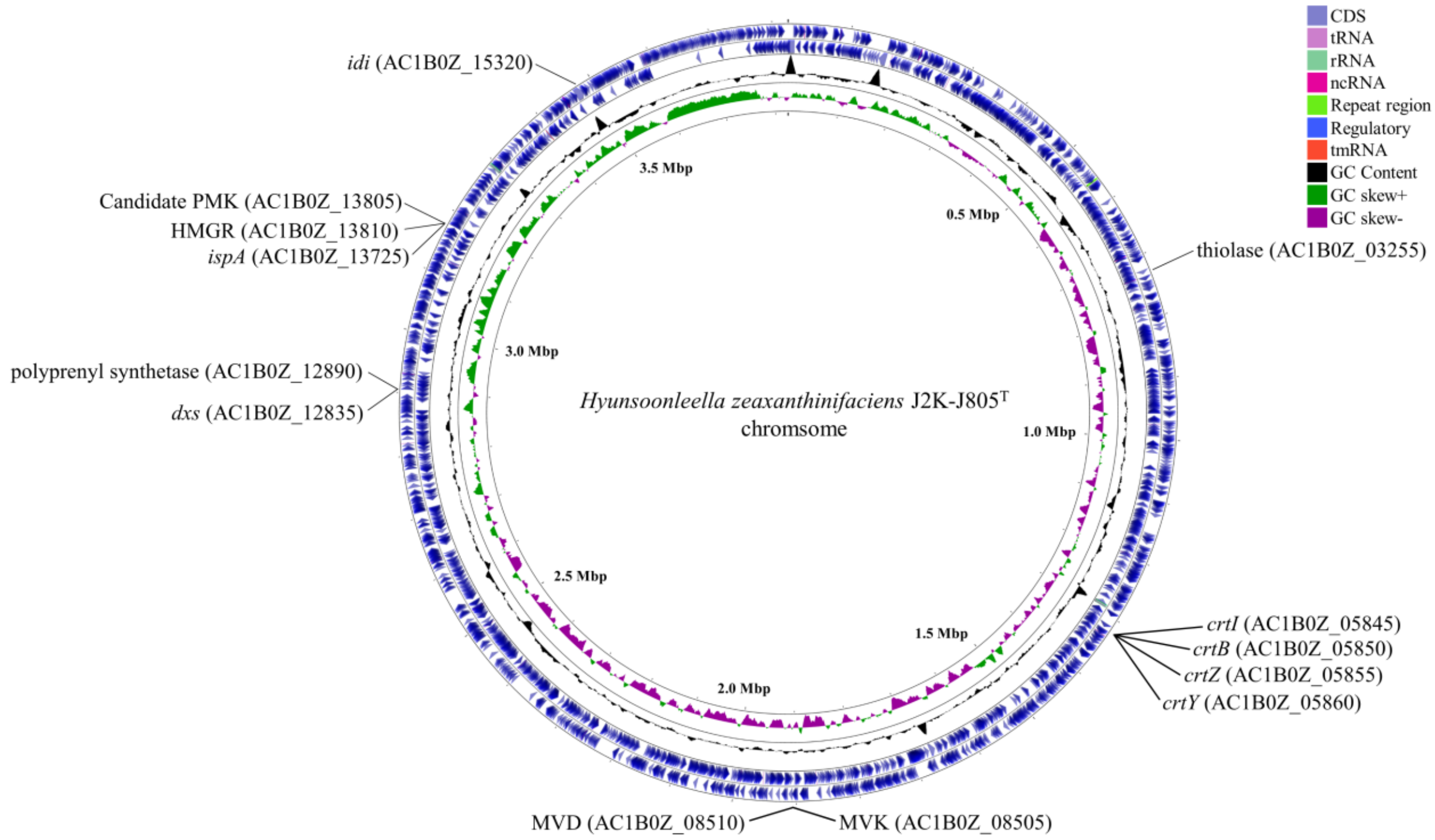

Fig. S2. Circular genome map of *Hyunsoonleella zeaxanthinifaciens* strain J2K-J805^{T}. Coding sequences, RNA genes and other annotated genomic features are shown in the outer tracks, whereas GC content and GC skew are shown in the inner tracks. Selected carotenoid- and isoprenoid-related genes are labelled. The detected *dxs*-like gene represents the first step of the MEP pathway, but downstream core MEP genes were not identified; therefore, this annotation was not interpreted as evidence for a complete MEP route.

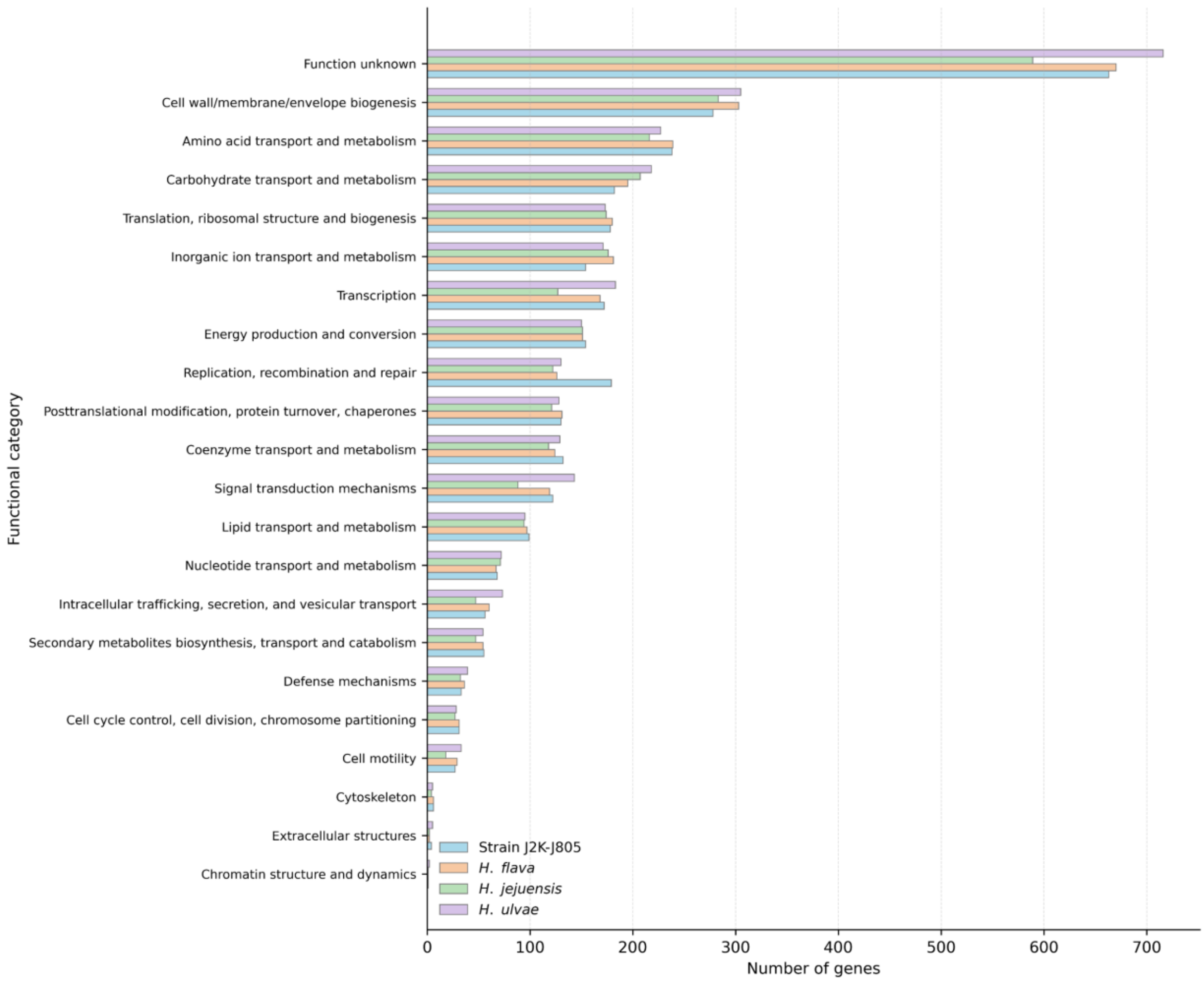

Fig. S3. COG functional category distribution of strain J2K-J805^T and related *Hyunsoonleella* type strains. Bars indicate the number of genes assigned to each functional category.

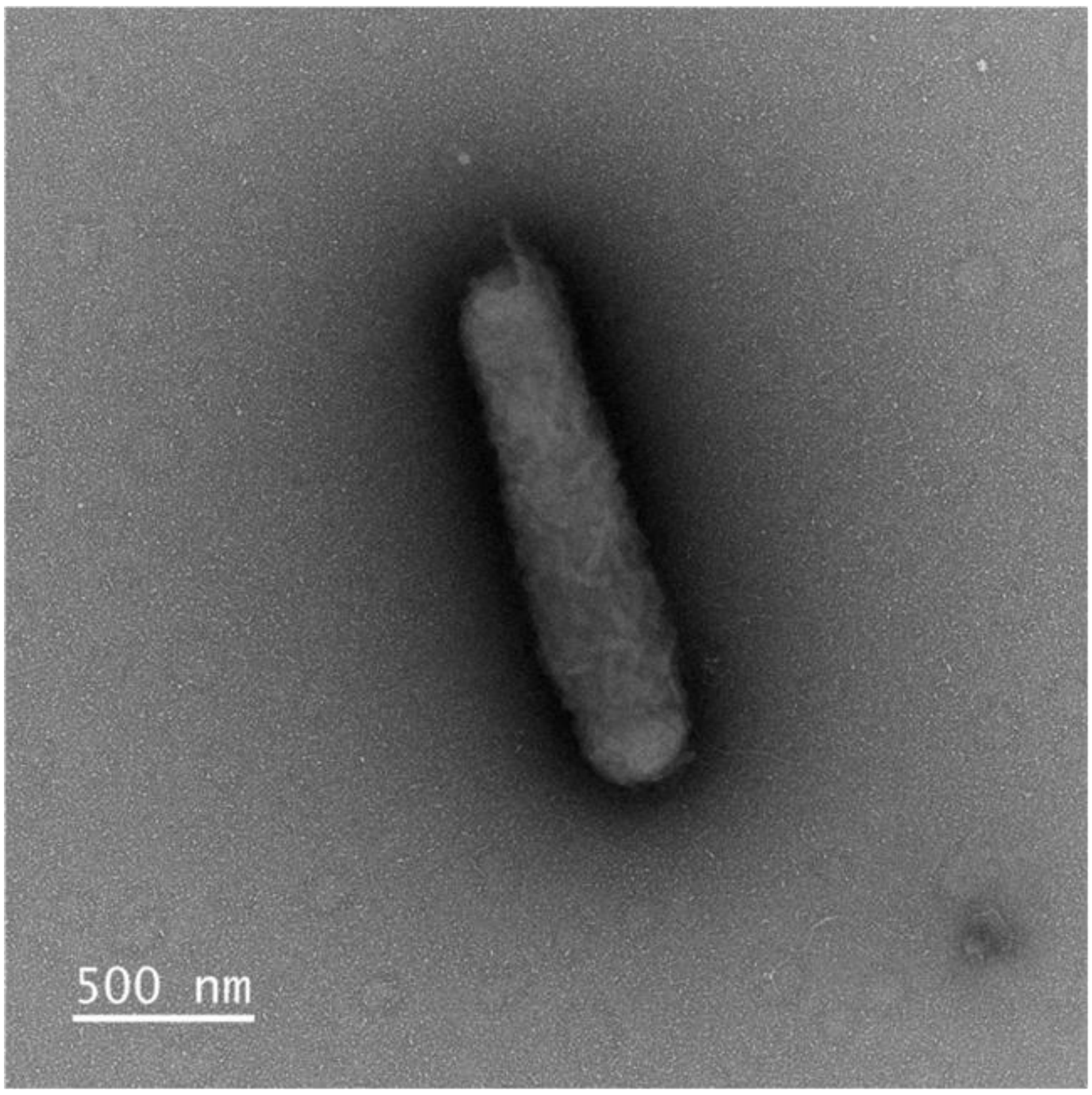

Fig. S4. Transmission electron micrograph of strain J2K-J805^{T}. Cells were observed after cultivation on marine agar at 25°C for 2 days. Scale bar, 500 nm.

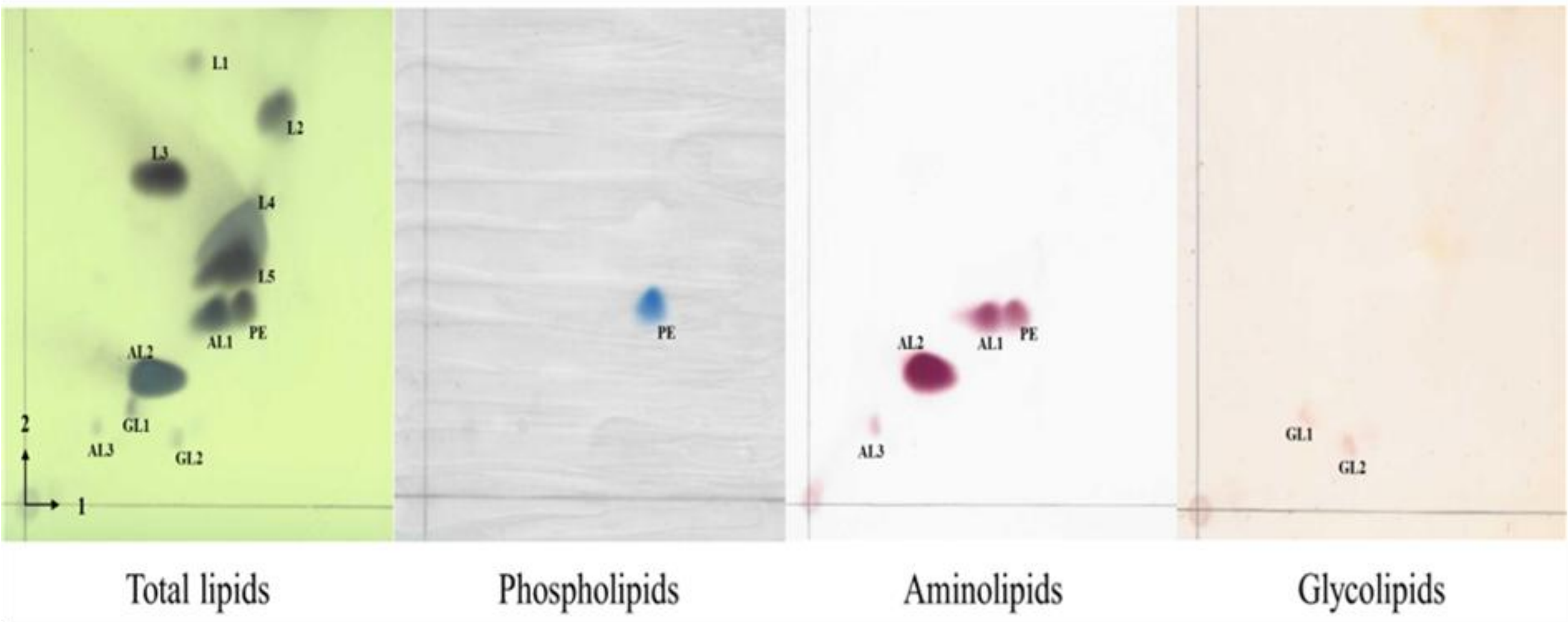

Fig. S5. Two-dimensional thin-layer chromatograms (TLC) showing the polar lipid profiles of strain J2K-J805^{T}. The solvent systems used were: (I) chloroform-methanol-water (65:25:4, v/v/v) and (II) chloroform-acetic acid-methanol-water (80:15:12:4, v/v/v/v). PE, phosphatidylethanolamine; AL, unidentified aminolipid; GL, unidentified glycolipid; L, unidentified lipid.

Table S1. Type-strain designations and available sequence accession numbers for the *Hyunsoonleella* taxa included in this study, non-*Hyunsoonleella* neighboring taxa shared between the 16S rRNA gene-based and phylogenomic analyses, and the outgroup.

**Species**	**Type strain designation(s)†**	**16S rRNA gene accession (Fig. 1, S1)**	**Genome accession (Fig. 2)**
Hyunsoonleella zeaxanthinifaciens	J2K-J805^{T} (= KCTC 102498^{T} = JCM 38469^{T})	PX861573	CM169901
H. flava	T58^{T} (= KCTC 72081^{T})	MK616102	SIRT01000001–SIRT01000063
H. jejuensis	CNU004^{T} (= KCTC 22242^{T} = DSM 21035^{T})	EF217419	FOFN01000001–FOFN01000011
H. pacifica	SW033^{T} (= CGMCC 1.11009^{T})	JN118553	BMEE01000001–BMEE01000013
H. rubra	FA042^{T} (= KCTC 42398^{T})	KR528480	JBHULY010000001–JBHULY010000047
H. aquatilis	SJ7^{T} (= KACC 21715^{T})	MN955402	JACNMF010000001–JACNMF010000021
H. udonensis	JG48^{T} (= KCTC 42341^{T})	KP271448	Not available (16S tree only)
H. aestuarii	KYW371^{T} (= KCTC 23449^{T} = JCM 17452^{T})	HQ405792	VAWB01000001–VAWB01000008
H. ulvae	HU1-3^{T} (= KCTC 82511^{T} = MCCC 1K05798^{T})	MW527409	JAEPJQ010000001–JAEPJQ010000023
Jejuia pallidilutea	EM39^{T} (= DSM 21165^{T})	EU443204	PVEO01000001–PVEO01000034
Seonamhaeicola algicola	Gy8^{T}	KR080554	VOSC01000001–VOSC01000034
Flavivirga rizhaonensis	RZ03^{T}	MK311254	SRSO01000001–SRSO01000104
Flavivirga jejuensis	JC2682^{T} (= KACC 14158^{T})	HM475139	JAUOEL010000001–JAUOEL010000033
Flavivirga eckloniae	ECD14^{T}	KU725769	CP025791
Algibacter luteus	P7-3-5^{T}	JQ807811	ALIH01000001–ALIH01000051
Flaviramulus multivorans	M-M16^{T}	KC618395	JAKKDV010000001–JAKKDV010000011
Pseudalgibacter alginicilyticus	HZ22^{T}	JN620360	CP012898
Brumimicrobium glaciale (outgroup)	ACAM 645^{T} (= IC156^{T} = CIP 107451^{T} = LMG 21434^{T})	AF521195	SETE01000001–SETE01000012

†Equivalent culture collection designations are provided to cross-reference taxa between the 16S rRNA gene-based trees (Fig. 1, Fig. S1) and the genome-based tree (Fig. 2); all shared taxa represent the same type strains.

Table S2. Pairwise OrthoANIu and digital DNA–DNA hybridization values between strain J2K-J805^T and related *Hyunsoonleella* type strains.

Query strain	Reference type strain	OrthoANIu (%)	dDDH (%, $d_0/d_4/d_6$)
Hyunsoonleella zeaxanthinifaciens J2K-J805^T	*H. flava* T58^T	87.72	52.9 / 34.5 / 48.1
	H. jejuensis DSM 21035^T	79.20	24.0 / 22.5 / 22.6
	H. ulvae HU1-3^T	78.70	25.9 / 22.1 / 24.0

OrthoANIu values were calculated using OrthoANIu, and dDDH values were obtained using GGDC. dDDH values based on formulae d0, d4 and d6 are shown. Formula d_4 values were used for species-level comparison in the main text because GGDC formula 2 is independent of genome length and is suitable for comparisons involving draft genome assemblies. All values obtained using formulae d_0, d_4 and d_6 were below the 70% species delineation threshold.

Table S3. Acids production from carbohydrates by strains J2K-J805^T and their closely related *Hyunsoonleella* type strains.

**Carbohydrate**	**1**	**2**	**3**	**4**
D-Arabinose, L-arabinose, D-ribose, L-xylose and D-fucose	–	+	–	–
D-Mannose, amygdalin and D-lactose	+	+	–	+
D-Sorbitol, D-trehalose and D-turanose	–	–	–	+
Salicin and D-melezitose	+	–	–	–
D-Cellobiose	+	+	+	–
D-Melibiose	+	+	–	–
D-Sucrose	–	+	–	+
Glycogen	+	–	+	+

Taxa: 1, strain J2K-J805^T; 2, *H. flava* T58^T (KCTC 72081^T); 3, *H. jejuensis* CNU004^T (KCTC 22242^T); 4. *H. ulvae* HU1-3^T (KCTC 82511^T). All data were obtained from this study. All strains produce acids from the following carbohydrates: D-xylose, D-galactose, D-glucose, L-rhamnose, esculin, D-maltose, starch, *β*-gentiobiose and potassium 5-ketogluconate. All strains do not produce acids from the following carbohydrates: glycerol, erythritol, D-adonitol, *β*-methyl-D-xylopyranoside, D-fructose, L-sorbose, dulcitol, inositol, D-mannitol, *α*-methyl-D-mannopyranoside, *α*-methyl-D-glucopyranoside, *N*-acetyl-D-glucosamine, arbutin, inulin, D-raffinose, xylitol, D-lyxose, D-tagatose, L-fucose, D-arabitol, L-arabitol, potassium gluconate and potassium 2-ketogluconate. Symbols: +, positive; –, negative.

Table S4. Cellular fatty acid compositions (%) of strain J2K-J805^{T} and their closely related *Hyunsoonleella* type strains.

Fatty acid	1	2	3	4
Saturated:				
$C_{16:0}$	2.5	2.3	2.3	3.6
$C_{18:0}$	–	–	–	tr
Unsaturated:				
$C_{15:1}$ $\omega 6c$	tr	–	tr	tr
$C_{15:1}$ iso G	**23.1**	**24.2**	**20.5**	**20.4**
$C_{15:1}$ anteiso A	3.4	3.4	5.0	4.3
$C_{16:1}$ iso G	tr	tr	tr	2.0
$C_{17:1}$ $\omega 6c$	tr	–	tr	–
$C_{17:1}$ anteiso $\omega 9c$	–	tr	tr	–
Branched:				
iso-$C_{15:0}$	**17.6**	**19.0**	**19.5**	**11.7**
iso-$C_{16:0}$	1.3	1.0	1.0	1.7
anteiso-$C_{13:0}$	–	tr	tr	tr
anteiso-$C_{15:0}$	3.2	5.3	6.5	5.6
Hydroxy:				
$C_{15:0}$ 2-OH	1.1	1.1	1.3	1.5
$C_{17:0}$ 2-OH	2.1	3.1	3.2	4.0
iso-$C_{15:0}$ 3-OH	8.5	6.8	7.8	5.5
iso-$C_{16:0}$ 3-OH	6.9	7.7	4.9	9.7
iso-$C_{17:0}$ 3-OH	**17.3**	**14.3**	**14.8**	**16.5**
Summed feature*:				
3 ($C_{16:1}$ $\omega 7c$ and/or $C_{16:1}$ $\omega 6c$)	6.3	6.0	5.5	7.7
9 ($C_{16:0}$ 10-methyl and/or iso-$C_{17:1}$ $\omega 9c$)	tr	tr	1.3	1.2

Taxa: 1, strain J2K-J805^{T}; 2, *H. flava* KCTC 72081^{T}; 3, *H. jejuensis* KCTC 22242^{T}; 4. *H. ulvae* KCTC 82511^{T}. All data were obtained from this study. Data are expressed as percentages of the total fatty acids, and fatty acids constituting less than 1.0% in all strains are not shown. Major components (> 10.0%) are highlighted in bold; symbols: tr, trace amount (<1.0%); –, not detected.

*Summed features represent groups of two or three fatty acids that cannot be separated by GLC with the MIDI system.

Table S5. HPLC-PDA analysis of zeaxanthin standard solutions and the methanolic pigment extract of strain J2K-J805^T.

Sample	Retention time (min)	Peak area
Strain J2K-J805^T extract	6.229	169696
Zeaxanthin standard 1 μg/L	6.204	27287
Zeaxanthin 5 μg/L	6.207	335570
Zeaxanthin 10 μg/L	6.215	1440290
Zeaxanthin 50 μg/L	6.228	4162246
Zeaxanthin 100 μg/L	6.229	9213603

The retention times of the standards increased slightly with concentration owing to a mass-load-dependent shift in peak apex. Peak area is a quantitative detector-response value and was not used for pigment identification. The major pigment of strain J2K-J805^T was identified as zeaxanthin on the basis of a retention time within the authentic-standard window (6.204–6.229 min) and a matching PDA absorption spectrum (maxima at ~450 and 475 nm), not by matching peak area to a specific standard concentration.
